# Establishment of a type 1 diabetes structured education programme suitable for Chinese patients: type 1 diabetes education in lifestyle and self adjustment (TELSA)

**DOI:** 10.1186/s12902-020-0514-9

**Published:** 2020-03-10

**Authors:** Yuting Xie, Fang Liu, Fansu Huang, Chunna Lan, Jia Guo, Jing He, Lezhi Li, Xia Li, Zhiguang Zhou

**Affiliations:** 1 Department of Metabolism and Endocrinology, The Second Xiangya Hospital, Central South University, No.139 Middle Renmin Road, Changsha, China; 2 National Clinical Research Center for Metabolic Diseases, and Key Laboratory of Diabetes Immunology, Ministry of Education, Changsha, China; 3 Clinic Nursing Teaching and Research Section, The Second Xiangya Hospital, Central South University, Changsha, China; 4 Department of Clinical Nutrition, The Second Xiangya Hospital, Central South University, Changsha, China; 5 Department of Rehabilitation, The Second Xiangya Hospital, Central South University, Changsha, China; 6 Xiangya School of Nursing, Central South University, Changsha, China; 7 Medical Psychological Center, The Second Xiangya Hospital, Central South University, and Medical Psychological Institute of Central South University, Changsha, China

**Keywords:** Type 1 diabetes, Structured education programme, Qualitative interviews, Delphi method

## Abstract

**Background:**

Various guidelines recommend that all adults diagnosed with type 1 diabetes (T1D) should be offered an evidence based, structured education programme (SEP) to optimize self-management care. China has a 13,000 annual increase in newly diagnosed T1D cases, of which 65% are adults. However, there is yet no validated SEP targeted to T1D patients in China. The purpose of this study is to establish a structured T1D self-management education programme—‘Type 1 Diabetes Education in Lifestyle and Self Adjustment’ (TELSA) that is adapted to medical and cultural practices in China.

**Methods:**

TELSA programme was developed based on the ADDIE model, following three steps: i) Semi-structured interviews were administered to 10 healthcare professionals (HCPs) and 13 T1D patients. Different topic guides, focusing on 4 dimensions including goals, contents, format of delivery, and quality assurance, were designed for either HCPs or patients. The interviews were recorded and analysed with thematic analysis. ii) Extracted themes were modified according to Delphi consultation. iii) Preliminary courses were conducted as pilot study to evaluate the effects of TELSA and optimization of the curriculum was finalized accordingly.

**Results:**

A total of 18 themes in 4 dimensions of the programme design were identified in the final version: i) goals: ‘behaviour modification’ and ‘outcome improvement’; ii) contents: ‘living with T1D’, ‘self-monitoring of blood glucose’, ‘knowing insulin’, ‘insulin dose adjustment’, ‘carbohydrates and carbohydrate counting’, ‘hypoglycaemia’, ‘complications of diabetes’, ‘managing psychological issues’, ‘physical activity’, and ‘question-and-answer’; iii) format: ‘multidisciplinary team combined with peer support’, ‘face-to-face education followed by remote learning’, and ‘2-day programme held on weekends’; and iv) quality assurance: ‘after-class quiz’, ‘patients’ feedback’, and ‘long-term evaluation on effectiveness’.

**Conclusions:**

A type 1 diabetes structured education programme in China was set up and shown to be applicable under local medical, social, and cultural environment.

**Trial registration:**

NCT03610984. Date of registration: August 2, 2018.

## Background

Type 1 diabetes (T1D) patients suffer from continuous loss of pancreatic β-cell function and need life-long exogenous insulin treatment. Adjusting insulin dose to keep glucose levels at target in a flexible daily routine remains an enormous challenge in T1D management. Diabetes self-management education (DSME) is critical for patients to achieve HbA1c targets, minimise hypoglycaemia, and optimise quality of life. Self-management training programmes such as dose adjustment for normal eating (DAFNE) and the diabetes teaching and treatment programmes (DTTP) have consistently demonstrated positive outcomes in diabetes control as well as quality of life in multiple studies [1–4]. Therefore, consensus has been reached across various guidelines that all adults diagnosed with T1D should be offered an evidence based, structured education programme (SEP) [5, 6]. An SEP is defined as ‘a planned and graded process that facilitates the knowledge, skills and ability for diabetes self-management and empowers individuals to live healthily, to maintain and improve their quality of life, and assume an active role in their diabetes care team.’ [5] It incorporates the needs, goals, and life experiences of patients [7, 8]. Five quality criteria of an SEP include i) patient-centered philosophy, ii) structured written curriculum, iii) trained educators, iv) quality assurance, and v) regular audit [5]. However, although structured education for T1D is an absolute necessity, only limited countries have established proper SEPs [7].

A recent study showed that China has 13,000 newly diagnosed T1D cases every year in spite of a low incidence rate [9], and is now the fourth largest country in the number of T1D cases in the 0–14 age group [10]. Even with expanding categories of insulin and new technologies, the mean HbA1c across all age groups reported in the cost, coverage, and care (3C) study in China remained as high as 8.9% [11]. Although only 35.2% of patients had eye exams, the percentage of patients that have been diagnosed with diabetic retinopathy reached 17.3% in their investigation [11]. The severe status quo on diabetes-related comorbidity is largely due to the lack of diabetes self-management education from routine clinical care [11]. To date, there is no evidence based and validated SEP available to T1D patients in China. The lack of standard T1D educational approaches has resulted in remarkable regional disparities in diabetes control as well as varied clinical practice routines across different healthcare settings.

Due to lifestyle and social differences, SEPs should be tailored to each country. There is an urgent unmet need for a high-quality, standard T1D curriculum that empowers Chinese T1D patients with self-management skills and psychological support. Hence this study focused on establishing the ‘Type 1 Diabetes Education in Lifestyle and Self Adjustment’ (TELSA) programme, the first T1D SEP designed to match the medical, social, and cultural environment in China.

## Methods

### Study design

The training programme was developed based on the ADDIE instructional design process [12–14]. Three essential steps: semi-structured interviews, nationwide Delphi consultation, and preliminary courses, were included in the development of TELSA.

### Part 1: Semi-structured interviews

#### Data collection

A qualitative, descriptive design was adopted. Healthcare professionals (HCPs) certified for T1D clinical care and adult T1D patients (≥18 years of age) were recruited with purposive sampling strategy as interviewees. Patients with diabetes duration ≥3 months and no medical history of mental disorders or cognition impairment were recruited from 2 tertiary hospitals in Beijing and Changsha. HCPs who were certified for T1D clinical care and familiar with diabetes education programmes were selected from 4 tertiary hospitals in Beijing, Xi’an, Suzhou, and Changsha. All interviewees gave their informed consent. Interviews were conducted by a diabetes education nurse. Interview guidelines for either patients or HCPs (Additional file 1) were created by the research team and piloted in two interviews (not included in the analysis) to assess comprehensibility and avoid content that could be misconstrued or offensive. Questions were open-ended and leading questions were avoided. Each interview lasted 30 to 60 min and was audio-recorded with participants’ consent. In the last two interviews in each group, no new information related to the topic was obtained, which suggested that saturation was achieved and data collection was ended [15]. The final sample consisted of 13 T1D patients and 10 HCPs (Tables 1 and 2).

**Table 1:** Characteristics of patients interviewed

Characteristics	Patients (*n* = 13)
**Gender, n (%)**	
Male	6 (46.2)
Female	7 (53.8)
**Age in years, mean (range)**	30.9 (19.0–52.0)
**T1D duration in years, mean (range)**	10.1 (0.5–41.0)
**Highest level of education, n (%)**	
Junior high	2 (15.4)
Senior high	1 (7.7)
College/University	7(53.8)
Postgraduate	3(23.1)
**Marital status, n (%)**	
Married	5 (38.5)
Single	8 (61.5)

**Table 2:** Characteristics of healthcare professionals interviewed

Characteristics	Professionals (*n* = 10)
**Gender, n (%)**	
Male	1 (10.0)
Female	9 (90.0)
**Age in years, mean (range)**	37.9 (30.0–53.0)
**Highest level of education, n (%)**	
Doctor	6(60.0)
Master	4(40.0)
**Professional, n (%)**	
Diabetologist	5 (50.0)
Diabetes specialist nurse	2 (20.0)
Diabetes educator	3 (30.0)
**Years of working, mean (range)**	14.4 (6.0–31.0)

#### Data analysis

The five stages of thematic analysis were followed [16]. Records were transcribed verbatim within 24 h of each interview by the interviewer and were re-read and verified. NVivo 11.0 (QSR International Pty Ltd) was used for data analysis. Inductive content analysis was performed [16]. The transcripts were then coded and themes were identified, followed by discussion and examination with a second researcher. Disagreements were discussed within the research team to aid refinement.

### Part 2: Delphi consultation

#### Participants

Experts were selected as potential participants if they are: i) recognized experts in the field of T1D or diabetes education, ii) researchers with expertise in diabetes, education, or psychology, identified through literature review, and iii) researchers recommended through experts’ professional network. Purposive sampling was conducted. Ultimately, a multidisciplinary and geographically diverse group of 25 Chinese-based experts were invited in this Delphi panel (Additional file 2).

#### Delphi procedure

The Delphi questionnaires were delivered to participants in person or via emails and responses were collected within two weeks. The questionnaire started with an introduction that briefly explained the purpose of the study, and included general information on participants. The main part of the questionnaire included 17 items developed from qualitative interviews. The five-point Likert scale was used to evaluate the degree of importance for each item (5 for ‘extremely important’ to 1 for ‘unimportant’). Participants’ basis of judgment and degree of familiarity for each item were also surveyed in this questionnaire. Free-text columns were included for collection of suggestions. Data were collated and re-presented to the research team for further refinement of TELSA. Here we defined a consensus regarding a specific item as at least 80% agreement (with Likert score 4–5) [17].

### Part 3: Preliminary courses

The first two courses were piloted to evaluate the acceptability and organization of the curriculum. Announcements of the courses were released in the WeChat (Tencent Corp) public group where all the registered T1D patients in our T1D clinic had access to. For each course, the first 10 adult patients volunteered to join were selected as attendees. After the course, all attendees completed feedback questionnaires and a quiz designed by the research team (Additional file 3). Patients were also encouraged to leave their free text comments. Questionnaires were presented in a multiple-choice format with free-text comments column. Questions covered degree of satisfaction, course comprehensibility, acceptability, and usefulness. The quiz contained at least 2 questions per session, with an emphasis on carbohydrate (carb) counting and insulin dose adjustment. Pre-testing of the quiz was conducted among researchers to avoid misunderstandings. Curriculum was reviewed and modified by the research team according to patients’ responses.

## Results

### Part 1: Semi-structured interviews

Totally 17 themes in the 4 dimensions of curriculum were identified.

#### Goals

##### Theme 1: Outcome improvement

All patients and HCPs ranked improving diabetes outcome, including both physiologically and psychologically, the most important goal of diabetes education. To live ‘a normal life’ or have ‘better quality of life’ is also of top concern.

*‘A qualified education programme is supposed to impact on patients’ metabolic control. It is critical in improving the quality of life for patients and their family.’*

*[Healthcare professional (H) 1].*

*‘I want to reduce the number of hypoglycaemic episode as well as incidences of very high blood sugar, and ultimately, to avoid complications.’*

*[Patient (P) 1].*

*‘What I hope is to be able to relieve my anxiety, and thus live a normal life.’*

*[P9].*

##### Theme 2: Behaviour modification

HCPs in China will sometimes encounter T1D patients admitted with diabetic ketosis due to discontinuation of insulin therapy, because of invalidated treatments that can ‘cure’ T1D. Moreover, patients are frequently confused with different insulin categories and risk purchasing a wrong cartridge. Therefore, most HCPs recognized the importance of educating patients to achieve sufficient basic knowledge to avoid unnecessary cost.

*‘The most important goal of education is to reduce fundamental errors. For instance, some patients choose invalidated Chinese traditional medicine instead of insulin in the hope of curing T1D; some patients misuse NovoRapid 30 Mix as bolus instead of NovoRapid. These can be avoided through proper education right after diagnosis.’*

*[H2].*

*‘Through education, patients will have a correct understanding of diabetes self-management. … With various online resources that might be misleading, patients need easy access to professional educational materials that can teach correct from incorrect.’*

*[H4].*

#### Contents

##### Theme 1: Living with T1D

HCPs all agreed that the teaching session should start with a basic introduction to T1D.

*‘In the very beginning patients need to clearly understand what they are dealing with every day. … They should know, for example, what causes T1D, and what some of the most common misunderstandings of T1D are.’*

*[H7].*

In addition to basic concepts, patients were generally more concerned about ‘to build a healthy attitude towards diabetes’ in the first place. 8 out of 13 patients mentioned an ice-breaking start with establishing their confidence to live with diabetes for the rest of their lives.

*‘I think in the first class, it should be clarified what the benefits are for controlling diabetes. It should be pointed out that we can still have a normal life if diabetes is under control, so as to build our confidence.’*

*[P2].*

*‘… To help us correctly recognize ourselves: people who will be living with T1D life-long.’*

*[P5].*

##### Theme 2: Self-monitoring of blood glucose (SMBG)

The importance of SMBG has been greatly underestimated by most patients [18]. Over 60% patients in our T1D clinic tested blood glucose levels less than 4 times per day (data unpublished). However, only a few patients in the interview mentioned SMBG.

*‘I started with testing my blood glucose levels at least 7 times per day in the hope that I could figure out my glucose pattern. But soon I found it was a mission impossible. Then I just gave up and let it be. Now I just test randomly. As long as it is neither too high nor too low, I’m satisfied.’*

*[P4].*

*‘I’ve heard there is a way to check my blood glucose levels without finger prick. I’d like to know more about that.’*

*[P10].*

Patients’ negligence on SMBG is exactly why HCPs emphasize this topic. New techniques for glucose testing such as continuous glucose monitoring also need to be introduced.

*‘In addition to the normal range, frequency, and correct procedures of testing blood glucose levels, the importance of SMBG should also be emphasized in different conditions, helping patients to form a habit of testing.’*

*[H7].*

##### Theme 3: Managing psychosocial stress

Almost all patients were stressed at different levels and occasions—stress in the process of schooling, working, and establishing relationships. Some patients have difficulties in communicating with people around them about diabetes.

*‘My previous research found that T1D patients are under great stress but generally don’t know how to relieve it, or to effectively communicate with families and friends to gain support.’*

*[H3].*

*‘Since I have been diagnosed with diabetes, I have not been in the mood for starting a romantic relationship. I strongly believe no one will accept a young man with diabetes as a boyfriend.’*

*[P4].*

*‘All my classmates know that I’m diabetic. Every time I inject insulin before a meal, I think they are pitying me. So I just hide. I don’t like discussing it with others.’*

*[P11].*

##### Theme 4: Insulin

Insulin is essential for blood glucose control in T1D. Both the dose and timing of bolus injections are key factors in controlling post-prandial glucose [19]. However, it is not easy for the patients to understand how different types of insulin work.

*‘I want to learn the effect (PK-PD) of different insulin, like the time of onset, peak time, and etc.’*

*[P6].*

*‘Patients need to know the basics of insulin, such as its physiological effects and classification. This is the prerequisite of administering correct insulin at correct time.’*

*[H7].*

HCPs also emphasize on self-adjustment of insulin doses in daily routine in order to help patients to maintain ‘a normal life’.

*‘Blood glucose levels may vary every day. Instead of fixed doses prescribed by the doctors, it’s more important for the patients to learn how to adjust insulin doses properly according to their own meals and activities.’*

*[H10].*

##### Theme 5: Carbohydrate and carb counting

Carbohydrates are the staple food in Chinese cuisine. Although cooking methods and ingredients are more diversified and harder to predict compared to western food culture, consensus was still reached that, along with insulin, identifying carbohydrates, knowing carb counting and carb-insulin ratio, are fundamental.

*‘Through understanding how carbohydrates and other ingredients affect blood glucose levels, patients can learn to inject boluses before meals or snacks. If they want to live a less restricted life while keeping stable blood glucose levels, carb counting is a basic skill.’*

*[H9].*

*‘I heard from other patients about reading food labels, but I still have no idea what to look at. Energy? Carbohydrates? What’s the meaning of reading these numbers anyway? I am so afraid of being in the supermarket now, dare not to buy anything.’*

*[P9].*

##### Theme 6: Hypoglycaemia

Hypoglycaemic events occur to every T1D patients, but not everyone knows the right procedure to handle it. All basic dimensions of hypoglycaemia need to be elucidated.

*‘In fact, quite a few patients don’t realize that they are correcting hypoglycaemia in a wrong way. They need to learn how to treat and prevent hypoglycaemia correctly.’*

*[H3].*

*‘Besides, we need to let them understand what causes low blood glucose levels.’*

*[H9].*

*‘I want to know how to reduce hypoglycaemic events.’*

*[P2].*

##### Theme 7: Physical activity

When it comes to the patients’ physical activity, the biggest obstacle is knowing the trend of glucose fluctuations during and after exercises. Some experienced repeated hypoglycaemia after household chores. Some didn’t know their glucose changes because they never checked before or after.

*‘I don’t know what type of exercise fits me. I assume exercise can lower blood sugar, but my sugar level can’t even drop for 1 mmol/L after running for 5 kilometres.’*

*[P1].*

*‘Physical activity definitely has profound effects on glucose levels thus should be discussed. For example, patients need to learn how glucose levels might fluctuate with different types, duration, and strength of activities; how to make certain adjustments on food intake and insulin dose before, during, and after physical activities.’*

*[H1].*

Patients also mentioned the lack of time, and more importantly, perseverance to exercise regularly.

*‘I know the benefits of regular exercises, but it’s really easier said than done. I am so exhausted to even move my legs after work every day. I do want to know whether there is a better way to set myself in motion.’*

*[P7].*

##### Theme 8: Complications of diabetes

Screening for complications is recommended by diabetes guidelines [5, 6]. A comprehensive introduction to diabetic complications in plain language should be available.

*‘There must be a session for (chronic) complications, especially for the screening part.’*

*[H4].*

*‘…How to prevent and detect early signs of diabetic complications.’*

*[H5].*

*‘If we could know what diabetic complications feel like, probably we would pay more attention to them.’*

*[P4].*

##### Theme 9: Question-and-answer

Participants considered an opportunity for further discussion with HCPs and experienced patients a good summary of the whole course.

*‘There need to be a part where patients can freely and directly ask whatever questions they have during the course.’*

*[H1].*

*‘Question & Answer must be included… To guarantee that each attendee can have at least one actual problem solved.’*

*[P5].*

#### Format of delivery

##### Theme 1: Multidisciplinary team combined with peer support

SEPs in other countries are mainly conducted by a team of one diabetes education nurse and one dietician. Nevertheless, most HCPs interviewed embraced a multidisciplinary team of educators, with psychologists and physical therapists included.

*‘A multidisciplinary team is essential. Different contents should be elucidated by specific specialists—diets by dieticians, dose adjustment by diabetologists, and so on.’*

*[H4].*

From patients’ perspectives, peer support was indispensable. Patients are more likely to gain strength from people in the same condition.

*‘Experiences from well-managed patients will be quite valuable. ‘Long illness makes the patient a good doctor’. We cannot learn those personal experiences from textbooks or professionals. Plus, they can inspire us to never lose hope.’*

*[P12].*

##### Theme 2: Face-to-face education followed by remote learning

Face-to-face group learning remains the most effective setting by all participants. However, different from the large learning groups commonly seen among T2D patients in China, ‘small-group education’ was preferred for T1D. Meanwhile, with the rapid development of mobile health (mHealth) technologies, diabetes remote learning apps has become a welcomed supplement.

*‘I prefer patients sitting together because we can learn from each other. However, I’m not good at memorizing things, so it would be very helpful if we can watch video recordings back home.’*

*[P4].*

*‘Small-group teaching followed by remote learning is better. Remote learning should be via video recordings. It is more direct and intuitive.’*

*[H8].*

##### Theme 3: 2- to 3-day programme held on weekends or holidays

Most interviewees agreed that the length of face-to-face education should not exceed three days.

*‘Weekends are good options. It won’t conflict with any work. Besides, I think it will be too much to digest if the course lasts for more than 3 full days.’*

*[P8].*

#### Quality assurance

Questions about quality assurance were only administered to HCPs. Three themes were considered useful for developing an assessment system: ‘After-class quiz’, ‘Patient’s feedback’, and ‘Long-term evaluation of effectiveness’. While the first two themes mainly aim at examining immediate knowledge capture, general acceptance by patients and comprehensibility of the programme, the third theme evaluates effectiveness to provide insights for further refinement of the programme.

*‘A quiz held right after class is the most straight forward way to test the acceptance and outcome of teaching. You could know from attendees’ responses how well they have learned during class, and what needs to be explained again.’*

*[H2].*

*‘An extremely important indicator is patients’ responses, such as their experiences, degree of satisfaction, and so forth.’*

*[H10].*

*‘I think the final evaluation needs to be thorough, including both biomedical and psychological outcomes.’*

*[H3, H5, H8].*

### Part 2: Delphi consultation

All 17 themes reached consensus and results are shown in Table 3. Therefore, all items were preserved and no more rounds of Delphi were performed. According to experts’ suggestions, the research group modified the curriculum by i) changing the Chinese title for each lesson to make the course more intriguing, ii) splitting ‘insulin’ to ‘knowing insulin’ and ‘insulin dose adjustment’, iii) adding knowledge on diabetic ketoacidosis in the session ‘complications of diabetes’, and iv) arranging the course as a 2-day weekend event.

**Table 3:** Results of Delphi consultation

Dimension	Item	Agreement ratio^a^ (%)	Full mark ratio (%)	Degree of importance		
				Mean	SD	CV
Goals	Behaviour modification	100.00	92.00	4.92	0.28	0.06
	Outcome improvement	100.00	96.00	4.88	0.37	0.08
Sessions	Living with T1D	96.00	80.00	4.60	0.63	0.13
	Self-monitoring of blood glucose	100.00	92.00	4.88	0.44	0.09
	Managing psychosocial stress	92.00	68.00	4.28	0.80	0.19
	Insulin	100.00	96.00	4.72	0.33	0.07
	Carbohydrates and carb counting	100.00	88.00	4.76	0.37	0.08
	Hypoglycaemia	100.00	84.00	4.72	0.54	0.12
	Physical activity	100.00	80.00	4.68	0.52	0.11
	Complications of diabetes	96.00	84.00	4.48	0.56	0.12
	Question-and-answer	96.00	72.00	4.52	0.82	0.18
Format	Multidisciplinary team combined with peer support	100.00	96.00	4.84	0.20	0.04
	Face-to-face education followed by remote learning	96.00	84.00	4.76	0.63	0.13
	2- to 3-day programme held on weekends or holidays	92.00	72.00	4.48	0.93	0.24
Quality assurance	After-class quiz	100.00	80.00	4.64	0.70	0.15
	Patients’ feedback	96.00	84.00	4.88	0.54	0.11
	Long-term evaluation on effectiveness	96.00	80.00	4.60	0.85	0.18

^a^Agreement ratio is the proportion of experts scoring the item 4–5 points with Likert scale

### Part 3: Preliminary courses

In total, 20 T1D patients (Additional file 4) attended and 3 family members audited the courses. Degree of satisfaction was 100%, and all attendees rated the programme as ‘Extremely helpful’. 13 patients expressed their gratitude for this programme in comments. The average score on the quiz was 70%, and most incorrect answers were in dose calculation. According to patients feedback, ‘managing psychosocial stress ’ was expanded to ‘managing psychological issues’, the teaching methods of ‘carbohydrate counting’ and ‘insulin dose adjustment’ were modified to improve comprehensibility, and lunch break was extended to 2 h. Class schedule is shown in Table S5 (Additional file 5). The final version of TELSA programme is shown in Table 4.

**Table 4:** Final version of TELSA

Dimension	Theme
Goals	1. Behaviour modification
	2. Outcome improvement
Sessions	1. Living with T1D
	2. Self-monitoring of blood glucose
	3. Managing psychological issues
	4. Knowing insulin
	5. Carbohydrates and carb counting
	6. Insulin dose adjustment
	7. Hypoglycaemia
	8. Physical activity
	9. Complications of diabetes
	10. Question-and-answer
Format	1. Multidisciplinary team combined with peer support
	2. Face-to-face education followed by remote learning
	3. 2-day programme held on weekends
Quality assurance	1. After-class quiz
	2. Patients’ feedback
	3. Long-term evaluation on effectiveness

## Discussion

DSME is an essential component of diabetes management, especially in T1D [20]. Unfortunately, few T1D patients in China receive systematic DSME [11] due to the lack of standardized, validated SEPs. Therefore, we developed the first programme specific to T1D education in China.

### Validity of the development process

The overall curriculum was based on the ADDIE (i.e. Analysis, Design, Development, Implementation, and Evaluation) framework, ‘a cyclic process that evolves over time and continuous throughout the instructional planning and implementation process’ [14]. ADDIE is widely used in the development of training programmes and can assist developers in instituting a learner-centered approach. In this study, we integrated three procedures employing the ADDIE model.

Semi-structured interview is a commonly used approach for data collection in qualitative researches [21]. Diverse social and cultural environment of patients must be taken into consideration for educational programme to be feasible and effective. Thus, data were collected from patients and HCPs living and working in China, taking into account their own social and cultural situation [22]. Additionally, the qualitative design of Part 1 helped us to understand patients’ perspectives and daily life.

The validity of results from interviews was further verified with Delphi method. Delphi consultation is a validated and widely used technique for obtaining a consensus [23, 24]. In our study, the participants were not chosen randomly but were representative of their professional specialty [25], and had extensive experience with diabetes or education, as the average years of working experience was about 18 years. In the preliminary courses, all attendees reported a high level of satisfaction with the programme, regardless of diabetes duration or the current level of self-management skills. This suggests that the programme is well-fit for Chinese culture and social environment. However, patient enrollment methods in the preliminary courses favored patients with stronger initiation and willingness to receive diabetes education, thus a more positive feedback to the course. Further studies with more representative, average T1D patients are needed to evaluate the generalizability of this study to a broader T1D patient population.

### Comparisons with prior SEPs and perspectives

Derived from DTTP in Dusseldorf, Germany [26, 27], structured education courses have been adapted and implemented internationally. The principle approach is a flexible intensive insulin therapy adjusted to the carbohydrate component of the meal and the blood glucose level informed by SMBG [28]. Nonetheless, education needs to be tailored to fit the local audience. In a study by Grant et al. [29], glucose diary and doctor question-and-answer sessions were not considered as core components by interviewed educators in UK. However, as was shown in various themes such as ‘SMBG’ from our interviews, the perspectives of patients were quite different from those of educators. Therefore, our study integrated patients’ actual opinions with HCPs’ professional knowledge to maintain intactness of the whole programme, and was designed for the current status of diabetes management in China.

Another aspect that gives rise to regional divergence is the intensity of course delivery. Most SEPs in the UK are either 5 consecutive days or over several weeks [29, 30]. PRIMAS and DTTP in Germany are held during a 6-week period with 2 sessions per week [3]. These formats are quite unpractical in China due to diverse working environment as well as challenges in commuting. TELSA is designed as a 2-day programme with 10 lessons, which has not yet been reported in other SEPs. A DAFNE course delivered 1 day a week for 5 consecutive weeks (5-week course) was shown to be equivalent with the traditional 1-week course in terms of biomedical and psychosocial outcomes [31]. A 2.5-day educational intervention delivered through 6 weeks had no significant improvement on HbA1c or severe hypoglycaemia, but did show improvements in diabetes treatment satisfaction and patient empowerment [32]. However, intensity of delivery will influence course attendance [33, 34], and there has been no research focusing on the social-economic aspect of SEPs. We believe the shorter the course is, the less barriers there will be for attendance and implementation [34]. Whether 2-day is sufficient for an informative education course needs further evaluations.

Regardless of course intensity, some education sessions, such as calculating insulin doses, are generally considered challenging by both educators and course participants [29]. Many participants struggle to sustain behavior changes over time [35]. Results of after-class quiz in our preliminary courses also indicated that it would require patients more efforts to master carbohydrate counting and insulin dose calculation. Hence long-term improvements in glucose control after attending SEPs is compromised. Additionally, although classic SEPs are all face-to-face group education, this format also becomes a barrier for attendance [36]. Therefore, remote learning was highly advocated in our study to provide sustained education. It also has been suggested that providing practical follow-up support will likely extend the benefits of SEPs [35, 37, 38]. In addition to traditional SEPs, new formats and technologies are worth exploring in the near future.

## Conclusions

We developed TELSA—the first SEP targeted at Chinese adult T1D patients, using semi-structured interviews and Delphi method. The course was piloted and shown to be applicable in the local medical, social, and cultural environment. We will further conduct randomized controlled trials to evaluate the effectiveness of the programme. Strategies aiming at post-course follow-up supports will also be integrated to enhance learning.

## Supplementary information

**Additional file 1: Table S1** Interview guideline for adult patients with T1D. **Table S2** Interview guideline for HCPs.

**Additional file 2: Table S3** Characteristics of participants in Delphi consultation.

**Additional file 3:** Quiz and a sample of feedback questionnaire.

**Additional file 4: Table S4** Characteristics of T1D patients in preliminary courses.

**Additional file 5: Table S5** Class schedule of TELSA.

## Data Availability

The datasets used and/or analysed during this study are available from the corresponding author on reasonable request.

## Abbreviations

SEP: Structured Education Programme
SMBG: Self-monitoring of blood glucose
T1D: Type 1 Diabetes
TELSA: Type 1 Diabetes Education in Lifestyle and Self Adjustment
