# Supplementary material for: Establishment of a type 1 diabetes structured education programme suitable for Chinese patients: type 1 diabetes education in lifestyle and self adjustment (TELSA)
Source: BMC Endocr Disord. 2020 Mar 10;20:37. doi: 10.1186/s12902-020-0514-9 (PMC7063731; doi:10.1186/s12902-020-0514-9)
Supplement: Supplementary file 1 — Additional file 1: Table S1 Interview guideline for adult patients with T1D. Table S2 Interview guideline for HCPs. [file 12902_2020_514_MOESM1_ESM.docx]

Table S1 Interview guideline for adult patients with T1D

| 1. | Have you ever received any form of self-management education for type 1 diabetes? |
| --- | --- |
| 2. | In what way do you normally acquire type 1 diabetes related knowledge, including self-management skills? |
| 3. | Do you have any difficulties, confusion, or conflicts in managing type 1 diabetes in your daily life? |
| 4. | Do you consider it necessary to open a self-management education programme for patients with type 1 diabetes? |
| 5. | What do you consider most necessary to be included in a type 1 diabetes education course? |
| 6. | What format and intensity of delivery do you prefer? |
| 7. | What do you consider a better time for holding a type 1 diabetes education course? |
| 8. | How many days do you prefer for a type 1 diabetes education course? |
| 9. | Through the education programme, what goals do you want to achieve? |

Table S2 Interview guideline for HCPs

| 1. | How do you understand structured education programme for type 1 diabetes? |
| --- | --- |
| 2. | Do you consider it necessary to open a self-management education programme for patients with type 1 diabetes? |
| 3. | What do you consider most necessary to be included in a type 1 diabetes education programme? |
| 4. | What course format do you consider most appropriate for a type 1 diabetes structured education programme? |
| 5. | What specialties should be included in the educators’ team? |
| 6. | How many days do you consider appropriate for one education course? |
| 7. | Do you have any suggestions or advices on designing the curriculum? |
| 8. | What do you think are the goals of structured education programme for type 1 diabetes? |
| 9. | How should the quality of education programme be evaluated and assured? |
