# Supplementary material for: Establishment of a type 1 diabetes structured education programme suitable for Chinese patients: type 1 diabetes education in lifestyle and self adjustment (TELSA)
Source: BMC Endocr Disord. 2020 Mar 10;20:37. doi: 10.1186/s12902-020-0514-9 (PMC7063731; doi:10.1186/s12902-020-0514-9)
Supplement: Supplementary file 2 — Additional file 2: Table S3 Characteristics of participants in Delphi consultation. [file 12902_2020_514_MOESM2_ESM.docx]

Table S3 Characteristics of participants in Delphi consultation, N=25

| Gender, n (%) |  |
| --- | --- |
| Male | 2 (8.0) |
| Female | 23 (92.0) |
| Age in years, mean (range) | 39.76 (26-54) |
| Highest level of education, n (%) |  |
| Doctor | 13 (52.0) |
| Master | 8 (32.0) |
| Bachelor | 4 (16.0) |
| Professional, n (%) |  |
| Diabetologist | 6 (24.0) |
| Diabetes specialist nurse | 9 (36.0) |
| Diabetes educator | 4 (16.0) |
| Educator | 2 (8.0) |
| Psychologist | 4 (16.0) |
| Years of working, mean (range) | 17.80 (6-37) |
