# Supplementary material for: Establishment of a type 1 diabetes structured education programme suitable for Chinese patients: type 1 diabetes education in lifestyle and self adjustment (TELSA)
Source: BMC Endocr Disord. 2020 Mar 10;20:37. doi: 10.1186/s12902-020-0514-9 (PMC7063731; doi:10.1186/s12902-020-0514-9)
Supplement: Supplementary file 3 — Additional file 3: Quiz and a sample of feedback questionnaire. [file 12902_2020_514_MOESM3_ESM.docx]

Quiz

1. How many times should you test your blood glucose levels per day?

(At least four time per day. Before meals and bedtime)

1. (1) What test can tell you your average level of blood glucose over the past two to three month? (HbA1c)
2. What is the target level of this test? (Generally 7.0%)
3. Under normal circumstances, how long can last outside the fridge? (No more than one month)
4. What places are inappropriate to store insulin? (Multiple choice)

A. Checked baggage B. Car refrigerator C. Vacuum cup

D. Car trunk E. Carry-on baggage

(AD)

1. Which is not true about reusing the needles for insulin rejection?

A. It may cause infection of injection site.

B. It may cause lipohypertrophy at injection site.

C. It may let air go into the cartridge and make the insulin inactive

D. It may cause pain, bleeding, and incorrect doses at injection

E. Each needle can be reused for at most 6 times.

(E)

1. Which foods do you need to calculate carbohydrate when doing carbohydrate counting? (Multiple choice)
2. Taro B. Spinach C. Lotus root D. Boiled egg E. Cake

(ACE)

1. Fill in the blanks with the right answer below.

A. The dose of insulin per serving of carbohydrate

B. By how much 1 unit of insulin will reduce your blood glucose

The insulin/carbohydrate ratio indicates：____

The insulin sensitivity factor indicates: ____

1. List three conditions that can lead to hypoglycaemia.
2. _________________________________
3. _________________________________
4. _________________________________
5. What are the best foods to treat hypoglycaemia immediately? (Multiple choice).

A. Chocolate B. Sugar C. Fruit juice D. Noodle E. Apple

(BC)

1. Which is the wrong step for treating a person with severe hypoglycaemia?
2. Help the patient lie on the side but not on the back to prevent choke
3. Injecting glucagon into the buttock, arm or thigh

C. Give the patient glucose intravenous infusion

D. Make every effort to feed sweetened water or food

E. Make emergency call and accompany the patient

(D)

1. Which exercise may increase your blood sugar level? (Multiple choice)

A. 5-kilometer jogging B. 100 meter sprint C. 45-minute yoga

D. Mopping the floor for 1 hour E. Lifting weight for 20 minutes

(BE)

1. Before taking exercise, what should you do?

A. Carry quick acting carbohydrates with me.

B. Check my blood glucose before exercise.

C. Eat some carbohydrates based on my blood glucose level and the type of exercise planned.

D. Nothing needs to be done. Just to check my blood glucose level before next meal.

E. If my blood glucose is above 14 mmol/L, I need to inject some rapid acting insulin before taking exercise.

(ABC)

1. When should you test your blood or urine ketones?
2. Hypoglycaemia occurs.

B. Having nausea or vomiting.

C. Blood glucose is persistently above 13.9mmol/L.

D. Feeling sick

(BCD).

1. Under normal circumstances, what should be screened annually?

A. Urine albumin B. HbA1c C. Foot screen D. Eye exams

(ACD)

1. According to the blood glucose levels and food diary, calculate the insulin dosage or carbohydrate amount, and fill in the blanks.

| Date | Time | 6:30 | 12:00 | 15:30 | 19:00 | 23:00 | Notes |
| --- | --- | --- | --- | --- | --- | --- | --- |
| Monday | Carbohydrate (g) | 60 | 85 | 25 | 80 | // | No exercise |
|  | Blood glucose level (mmol/L) | 6.6 | 8.3 | 5.3 | 7.8 | 9.7 |  |
|  | Rapid-acting insulin doses | 4 | 6 | 2 | 6 | // |  |
|  | Long-acting insulin doses | // | // | // | // | 17 |  |
| Tuesday | Carbohydrate (g) | 60 | 90 | 20 | 80 | // | Started jogging at 15:30 for 40 minutes |
|  | Blood glucose level (mmol/L) | 5.9 | 3.8 | 7.1 | 11 | 10.3 |  |
|  | Rapid-acting insulin doses | 4 | ① | ② | ③ | // |  |
|  | Long-acting insulin doses | // | // | // | // | 17 |  |
| Wednesday | Carbohydrate (g) | 65 | 90 | ④ | 85 | // | No exercise and ate 25 grams of soda crackers at 15:30. |
|  | Blood glucose level (mmol/L) | 5.2 | 4.1 | 8.2 | 15.8 | 11 |  |
|  | Rapid-acting insulin doses | 4.5-5 | ⑤ | No insulin | ⑥ | // |  |
|  | Long-acting insulin doses | // | // | // | // | 17 |  |

①5 ②0 or 1 ③8 ④15 ⑤7 ⑥9

**Feedback Questionnaire**

**Session：Hypoglycaemia**

1. Generally, are you satisfied with this session?

1）Very satisfied □ 2）Satisfied □ 3）Neutral □ 4）Dissatisfied □

1. Is this session comprehensible？

1）Yes, very easy to understand □

2）Generally easy to understand, except for very few hard points □

3）Generally a little hard for me to understand □

4）No, too hard for me to understand □

1. How do you like the format of teaching in this session?

1）Very much □ 2）Generally good □ 3）Neutral □ 4）Dislike □

1. How do you like the classroom environment during this session?

1）Very much. I can express myself freely □

2）Generally good □

3）Neutral □

4）No. I do not want to talk □

1. Is the content in this session helpful to you?

1）Very helpful □ 2）Generally helpful □

3）Neutral □ 4）Not helpful at all □

1. For the main contents (①～④) of this session, please write down the number for each question (multiple choice).

①The definition of hypoglycaemia ②The harm of hypoglycaemia

③How to treat hypoglycaemia ④How to prevent hypoglycaemia

1) Which contents are the most helpful to you?

2) Which contents are the least helpful to you?

3) Which contents are not comprehensible?

4) Which contents are the most impressive?

7. Are there other aspects of hypoglycaemia that you don’t understand and are not covered in this session?

1）No □

2）Yes □ Please list：

8. Please also leave your comments of this session or any suggestions for improving the quality of this session.
