# Supplementary material for: Establishment of a type 1 diabetes structured education programme suitable for Chinese patients: type 1 diabetes education in lifestyle and self adjustment (TELSA)
Source: BMC Endocr Disord. 2020 Mar 10;20:37. doi: 10.1186/s12902-020-0514-9 (PMC7063731; doi:10.1186/s12902-020-0514-9)
Supplement: Supplementary file 4 — Additional file 4: Table S4 Characteristics of T1D patients in preliminary courses. [file 12902_2020_514_MOESM4_ESM.docx]

Table S4 Characteristics of T1D patients in preliminary courses, N=20

| Gender, n (%) |  |
| --- | --- |
| Male | 9 (45.0) |
| Female | 11 (55.0) |
| Age in years, mean (range) | 33.9 (19.0-49.0) |
| T1D duration in years, mean (range) | 5.9 (0.3-25.0) |
| Highest level of education, n (%) |  |
| Junior high | 2 (10.0) |
| Senior high | 4 (20.0) |
| Associate degree | 10 (50.0) |
| Bachelor and higher | 4 (20.0) |
| HbA1c, mean (range) | 8.2 (5.7-12.4) |
