# Supplementary material for: Establishment of a type 1 diabetes structured education programme suitable for Chinese patients: type 1 diabetes education in lifestyle and self adjustment (TELSA)
Source: BMC Endocr Disord. 2020 Mar 10;20:37. doi: 10.1186/s12902-020-0514-9 (PMC7063731; doi:10.1186/s12902-020-0514-9)
Supplement: Supplementary file 5 — Additional file 5: Table S5 Class schedule of TELSA. [file 12902_2020_514_MOESM5_ESM.docx]

Table S5 Class schedule of TELSA

| **Day 1** |  | **Day 2** |  |
| --- | --- | --- | --- |
| Time | Session | Time | Session |
| 8.00-8.30 | Warm up-Introduction | 8.00-9.00 | Individual dose adjustment |
| 8.30-9.30 | Living with T1D | 9.00-10.00 | Hypoglycaemia |
| 9.45-10.45 | Self-monitoring of blood glucose | 10.15-11.15 | Managing psychological issues |
| 10.45-11.45 | Knowing insulin | 11.15-12.00 | Complications of diabetes |
| 12.00-14.00 | Lunch break | 12.00-14.00 | Lunch break |
| 14.00-15.30 | Carbohydrate and carb counting | 14.00-15.00 | Physical activity |
| 15.30-17.00 | Insulin dose adjustment | 15.15-16.00 | Question-and-answer |
| 17.00-19.00 | Dinner, free chat | 16.15-17.00 | Quiz and questionnaires |
